# Comparison of surgical plume among laparoscopic ultrasonic dissectors using a real-time digital quantitative technology

**DOI:** 10.1007/s00464-012-2351-z

**Published:** 2012-06-04

**Authors:** Fernando J. Kim, David Sehrt, Alexandre Pompeo, Wilson R. Molina

**Affiliations:** 1 Division of Urology, Denver Health Medical Center, 777 Bannock Street, MC0206, Denver, CO 80204 USA; 2 Division of Urology, University of Colorado Health Sciences Center, Aurora, CO USA

**Keywords:** ACE, Cordless, Ergonomics, ImageJ, Laparoscopy, New technology, Plume, Sonicision, SonoSurg, Ultrasonic dissector

## Abstract

**Background:**

This study aimed to analyze the effect of surgical plume generation from various ultrasonic dissectors on laparoscopic visibility, including the first cordless ultrasonic dissector, using a novel real-time digital quantification technique.

**Methods:**

The Covidien Cordless Sonicision, the Harmonic ACE, and the Olympus SonoSurg were applied to bovine liver with industry-specified settings. Consecutive activations were digitally captured from a laparoscope positioned to replicate the clinical setting. Plume was recognized by ImageJ software, and the percentage of pixels containing plume in each video frame was calculated. Analysis of variance statistical multi-analysis and Welch’s *t* test were computed for all *p* values.

**Results:**

The average maximum plume produced by the Sonicision, ACE, and SonoSurg with the maximum setting were respectively 8.76 % (range, 4.32–17.41 %), 18.04 % (range, 9.07–55.12 %), and 9.46 % (range, 5.68–22.12 %) (*p* = 0.026). The deviations between the ACE and the other devices were significant (*p* < 0.05). The average maximum plumes produced with the coagulation setting were 4.80 % (range, 0.24–19.83 %) for the Sonicision, 26.63 % (range, 8.12–73.50 %) for the ACE, and 0.21 % (range, 0.06–1.05 %) for the SonoSurg (*p* < 0.001). The differences between all the instruments in the coagulation setting were significant.

**Conclusion:**

To the authors’ knowledge, this is the first report on a real-time digital analysis of surgical plume generation using ImageJ software. In the coagulation setting, the SonoSurg generated minimal plume. The Sonicision obstructed approximately 4 %, whereas the ACE generated plume that obstructed 25 % of the laparoscopic field. In the cutting setting, the SonoSurg and Sonicision generated the least obstruction, whereas the ACE caused the most obstruction.

Ultrasonic technology relies on heat and pressure from an oscillating piezoelectric transducer coupled with a titanium blade to cut tissue and coagulate blood vessels, generating plume and elevation of device and tissue temperature [1, 2]. The activation of ultrasonic dissectors in laparoscopy generates plume obstructing vision in the laparoscopic field of view [3, 4]. Plume generation may interrupt surgery until its settlement or evacuation from the environment. Occasionally, plume can adhere to the laparoscope, requiring removal of the instrument with subsequent loss of intracavity vision, thereby demanding additional attention during laparoscopy. An understanding of how plume limits vision will improve efficiency in laparoscopic surgery.

The current study introduced a novel approach to direct measurement of the amount of plume obstructing the laparoscopic visual field. We collected data regarding the quantity of plume and compared the results between the different laparoscopic ultrasonic dissectors.

## Materials and methods

### Instruments

The Harmonic ACE (Ethicon Endo-Surgery Inc., Cincinnati, OH, USA), Olympus SonoSurg (Olympus USA, Center Valley, PA, USA), and first-generation cordless Covidien Sonicision (Covidien, Mansfield, MA, USA) were applied to bovine liver ex vivo with the maximum and industry-specified coagulation settings. Activations were captured and analyzed with ImageJ software (National Institute of Health, Bethesda, MD).

### Experimental model

**Fig. 1:**
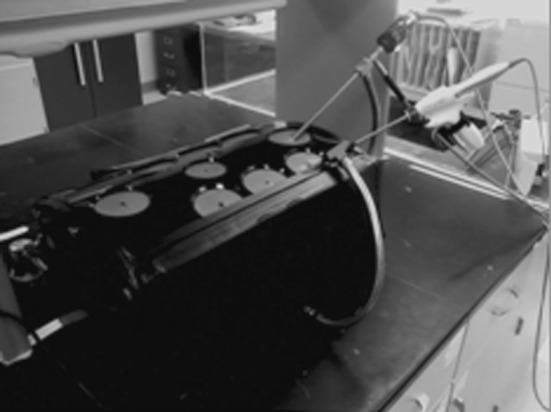
Experimental model

We modified a laparoscopic pelvic trainer (Karl Storz Szabo-Berci, Tuttlingen, Germany) to seal all sides of the box (Fig. 1). A side door was constructed to introduce the tissue into the system and was closed afterward. A black cloth was placed in the interior of the trainer to minimize reflection of light in the system and to enhance the contrast of plume.

An Olympus 10-mm scope was introduced into the top port and clamped in place. The scope was placed at approximately 35° to the bottom of the trainer directed at the opposite wall. The scope connected to an Olympus Viscera system, and imaging was recorded on an Olympus N-Stream.

An ultrasonic dissector was introduced into the remaining port on the top of the trainer and likewise clamped in place. The dissector also was placed at 35° to the bottom surface, with the tip centered in the laparoscopic field and located 10 cm from the tip of the scope. Tissue was introduced into the chamber, and the dissectors were activated for 2 and 3 s at industry-specified cutting and coagulation settings, respectively.

### Digital analysis

Consecutive activations were captured digitally in real time from the laparoscope positioned to mimic the clinical setting. Video imaging was imported into the ImageJ software to identify the area of plume frame by frame. Isolating plume from the video involved converting the red, green, and blue (RGB) image to an 8-bit black and white image. A filtering algorithm was applied twice to localize plume. The first filter was a pixel-intensity filter that passed an intensity value exceeding 6/255 (0 = black, 255 = white). The second was a spatial filter for objects greater than 5 pixels in area.

**Fig. 2:**
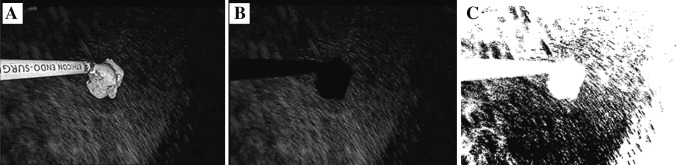
Digital analysis. **A** Plume recorded by the laparoscope. **B** Removal of the instrument and tissue. **C** Isolation and recognition of plume

This algorithm was first applied to remove the dissector and then to identify plume. The results are shown in Fig. 2. The number of pixels that contained plume then was counted in each frame and used to find the percentage of plume in the field. We measured an average background noise level of 0.05 % with this filtering technique.

### Statistical analysis

Descriptive statistics were produced with the R Project version 2.11 (The R Foundation for Statistical Computing, Wein, Australia). The percentage of plume was used to calculate the average plume versus time and maximum plume per activation. Data are presented as mean ± standard error unless otherwise noted. Analysis of variance (ANOVA) statistical multi-analysis and Welch’s *t* test were computed for all *p* values. A *p* value lower than 0.05 was considered significant.

## Results

**Fig. 3:**
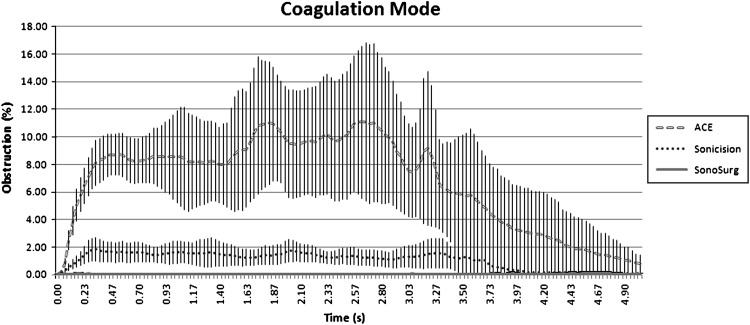
Average plume obstruction in coagulation mode versus time with confidence intervals

Table 1 summarizes the maximum plume obstruction from each trial. The SonoSurg produced minimal obstruction during activation. The ACE generated the most plume, with approximately five times more plume than the Sonicision. The maximum obstruction was in the range of 1.05 % from the SonoSurg to 73.50 % from the ACE. The differences between all the instruments in the coagulation setting were significant (*p* < 0.001). The average plume with respect to time was calculated with 95 % confidence intervals, as shown in Fig. 3. Likewise, the ACE generated the most plume obstruction of the three devices, whereas the SonoSurg had the least plume during coagulation activation.

**Table 1:** Maximum plume obstruction

Average coagulation obstruction	ACE	Sonicision	SonoSurg	*p* Value
Maximum obstruction (%)	26.63 ± 3.70	4.80 ± 0.86	0.21 ± 0.07	<0.001
Range (%)	8.12–73.50	0.24–19.83	0.06–1.05	
Average cutting obstruction				
Maximum obstruction (%)	12.65 ± 0.97	8.76 ± 1.49	9.46 ± 1.36	0.026
Range (%)	9.07–18.15	4.32–17.41	5.68–22.12

**Fig. 4:**
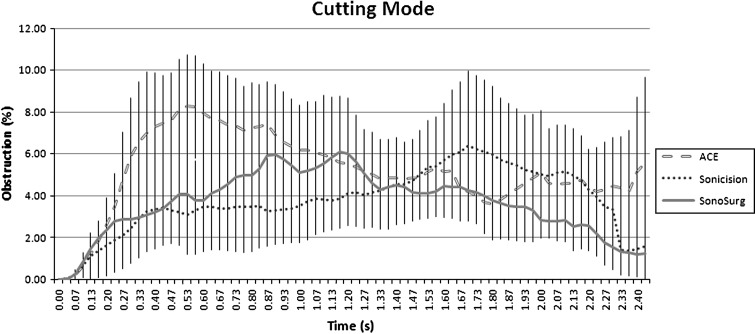
Average plume obstruction in cutting mode versus time with confidence intervals

There was less difference between the devices in the cutting mode. The Sonicision and SonoSurg produced the least amount of obstruction. Deviation of the ACE from the Sonicision and SonoSurg was significant (*p* < 0.05). Figure 4 shows the average maximum plume produced against time. The 95 % confidence intervals overlapped at all times with the other devices at the beginning of activation, except for the ACE.

## Discussion

Visualization of the surgical field in laparoscopic surgery is critical for successful outcomes. Laparoscopy has proved to be more difficult physically and psychologically for surgeons than open surgery [5, 6]. These stressors can be attributed to several factors including obstructed vision, rigid body positioning, and the counterintuitive movement of instruments [7, 8]. The process of settling and evacuating plume increases the workload during laparoscopy, which will fatigue, stress, and frustrate surgeons and prolong the operative time. Instruments producing minimal plume are highly sought after to facilitate laparoscopy.

A recent study analyzing surgical plume discovered that different types of energy-based instruments created different sizes and concentrations of plume particles. The differences in particle size and concentration were then hypothesized to affect visibility using the Rayleigh and Mie light scattering theories [9]. It was concluded that smaller mode particles with higher concentrations remained in suspension longer, which increased the obstruction of plume.

The geometric mean size of the small mode was 68.3 nm, and the size of the large mode was 994 nm, with respective concentrations of 6.10 × 10^5^ and 1.48 × 10^3^ particles/cm^3^. The size of the particles in the plume was seen to influence the obstruction, but this effect probably had little impact in our study because all the instruments applied ultrasonic technology.

The concentration of particles, on the other hand, could influence the discrepancy seen between devices. Although the mass generated from each activation was not measured, intuitively, more obstruction could suggest a higher concentration of plume.

Obstruction also may be affected by the pattern of plume emission. Plume generation appears to have two modes of emission: laminar and turbulent. Laminar emission occurs when there is constant pressure from the generating source, and plume formation appears conical in shape [10]. Turbulent emission is the result of rapid changes in pressure and velocity from the blade, which gives plume an irregular appearance. Comparing these two types of emissions, laminar emission minimizes the effect of plume on laparoscopic vision. The particles from laminar emission are located within a packet directed from the blade downward and settle on the cavity floor. Turbulent emission, on the other hand, produces particles that spread across the field with momentum in a broader range of directions. Turbulent plume with lateral and upward velocity takes longer to settle out of vision. The blade shape and operational consistency may influence this factor.

The demand for imaging analysis spawned ImageJ, an open-source image-processing program initially developed by the Research Services Branch of the National Institute of Health [11]. This software offers a number of valuable tools such as spatial and color filters, object edge identification, measurement tools, and statistical analysis. Its application has been directed primarily toward medical imaging and microscopy, but we have successfully applied this software to recognize objects in the laparoscopic field [12–14].

Future innovation in surgery may involve outlining and enhancing of anatomic structures on laparoscopic imaging, in vivo identification of tissue pathology, and removal of obstructing objects such as plume and instrument shafts. Image analysis also may have a role in education to assist observing students or to improve laparoscopic and robotic surgical simulators.

The ex vivo study design offered several advantages over an in vivo setting. The closed environment reduced any influence on the natural movement of plume. We admit that the laparoscopic environment deviates from our study system because insufflation introduces carbon dioxide into the cavity, and smoke evacuators also may be implemented. In addition, limited upward tension was placed on the tissue compared with surgical use, but this pressure remained consistent between devices.

The positioning of the instruments resembled the optimal ergonomics used in the operating room, although the study was limited to the most common orientation of the dissectors. Tissue selection was based on the moisture capacity of liver, but this liver was avascular and not optimal for coagulation. Although ex vivo use will differ from the in vivo environment, all the instruments were treated in a standardize fashion to control for variables that influence plume generation.

## Conclusion

To our knowledge, this is the first report on a real-time digital analysis of the quantification of surgical plume generation. Moreover, ImageJ allows surgeons to capture and analyze surgical plume in a variety of ways that may help the future design of laparoscopic instrumentation. The devices studied exhibited different degrees of plume production according to their maximum and coagulation settings. In the coagulation setting, the SonoSurg generated negligible plume. The Sonicision generated limited obstruction, whereas the ACE generated plume that obstructed one-fourth of the laparoscopic field. In the cutting setting, the SonoSurg and Sonicision generated the least obstruction, whereas the ACE generated the most.
